# Supplementary material for: Modeling and simulation of complex dynamic musculoskeletal architectures
Source: Nat Commun. 2019 Oct 23;10:4825. doi: 10.1038/s41467-019-12759-5 (PMC6811595; doi:10.1038/s41467-019-12759-5)
Supplement: Supplementary file 1 — Supplementary Information [file 41467_2019_12759_MOESM1_ESM.pdf]

Supplementary information for modeling and simulation of complex dynamic musculoskeletal architectures

Zhang *et al.*

Supplementary Note 1 — Anatomical modeling of muscles, tendons and bones

We are interested in capturing both biomechanics and morphology in musculoskeletal systems, whereby the Hill model and Cosserat theory are used. In addressing the biomechanical aspects, the Hill model interprets skeletal muscles as viscoelastic materials consisting of contractile elements (*CE*, myosin and actin), parallel elastic elements (*PE*, tissues wrapping around muscle fibers) and serial elastic elements (*SE*, tendons) (*1*) represented as dampers and springs (Supplementary Fig. 1a). The model has been widely used in kinesiological problems (*2, 3*) due to its simplicity. However, treating an entire muscle bundle as one integrated system results in the inability to selectively activate motor units of different sizes for fine-tuned motor control, to render individual motor units passive in the case of muscle injuries for customized rehabilitation or to account for the muscle's own deformation in response to system dynamics.

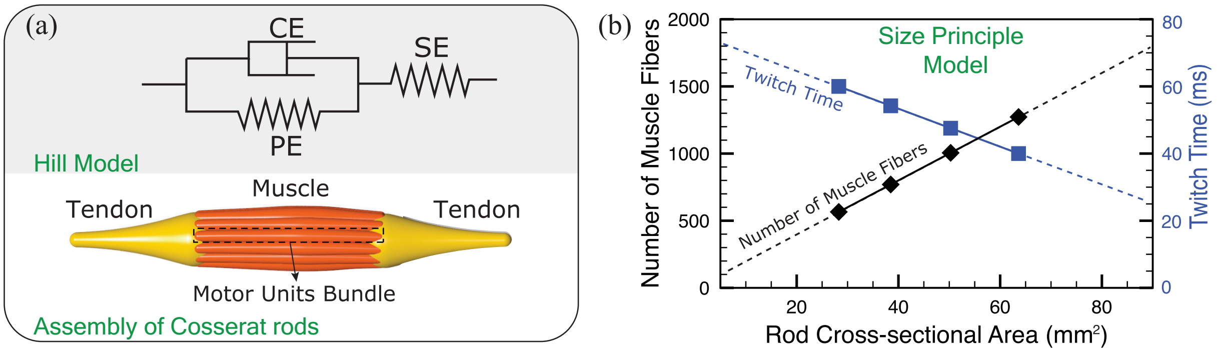

Supplementary Figure 1. **(a)** Hill three-element model—contractile (CE), parallel elastic (PE) and serial elastic (SE) elements—and simulation of a head of biceps brachii containing 18 filaments, each made up of 20 motor units. **(b)** Size principle relating muscle twitch time and peak force to motor unit size, based on (*4*).

Instead, we treat muscles as a collection of viscoelastic filaments and model each filament to represent motor units made of muscle fibers ($\leq 100\mu$m in diameter, which are never controlled individually). The number of fibers per filament depends on the desired level of actuation precision, the muscle type (*5*) and the individual (*6*). Here we relate the filament's cross-sectional area to its peak contraction force and twitch time via the *size principle* (*1*), for which low-force, slow-twitch are associated to small motor units (filaments), while high-force, fast-twitch to larger ones (Supplementary Fig. 1b). We assume a constant maximum voluntary contraction stress (σ_{MVC}) across the filament, forming a linear relationship between peak force output and motor unit cross-sectional area as suggested in (*4*). To relate size to twitch time, we consider a linearly decreasing relationship (*7*) with extreme values taken from (*1*). The mechanical properties of the filament, mainly elasticity and internal dissipation, the analogs of *PE* and *CE* respectively, are also based on biological data.

The replication of the two-headed muscle of an elbow joint, the biceps brachii (Supplementary Fig. 1a), may provide an intuition of our muscle modeling approach. Each head is made of 360 motor units based on (*8*) and equally-distributed among $N = 18$ filaments. While the num-

ber of motor units is a physiological, well-defined quantity, their grouping into 18 contractile model filaments that can be autonomously recruited is a modeling approximation. The level of course-graining can be adjusted depending on the desired level of detail and can even match all 360 physical units, although this will increase computational costs. Relating filament diameter to muscle fiber count in each motor unit (assuming an average fiber diameter of 75 μm (*9*)), we create a biceps head with a cross-sectional area $A = 804.2$ mm^2 and 18×10^4 muscle fibers, approximately consistent with the cross-sectional area and muscle fiber count reported in (*9*). Generalizing this approach allows us to replicate any generic muscle bundle capable of multi-level muscle recruitment through the appropriate combination of filament diameters and motor unit counts in each filament. For all the study cases presented in this paper, we assume the muscle unit's behavior is modeled as established here, unless specified otherwise.

Tendons and bones are instead represented as passive slender filaments with tapering segmental radii, whose biomechanical properties are also set according to experimental data. With these basic building blocks (muscles, tendons and bones) in hand, we are capable of replicating complex musculoskeletal systems given their biomechanical properties, as long as they are comprised of slender elements.

Supplementary Note 2 — Model validation: the human elbow joint

In order to validate our numerical approach, we consider the human elbow joint comprised of muscles, tendons and bones (Main Fig. 1a). We model the biceps brachii described in Supplementary Note 1. The bones (humerus, ulna and radius) are modeled as passive, rigid rods whereas the tendons (proximal and distal) are modeled as tapered passive but elastic rods, both with tapering geometrical features. The final assembly of the elbow joint is achieved through the boundary conditions described in the Methods as well as the human elbow joint section in the main text. The biomechanical properties of each element are detailed in Supplementary Table 1.

Supplementary Table 1. Bio-mechanical properties of muscles, tendons and bones used in simulations.

**Parameters**	**Values**	**Parameters**	**Values**
Muscle E (*10*)	10 kPa	Muscle Density (*11*)	1.06 g cm^{-3}
Bone E (*12*)	15 GPa	Bone Density (*12*)	1.75 g cm^{-3}
Tendon E (*13*)	500 MPa	Tendon Density (*13*)	1.67 g cm^{-3}
Humerus Length[a]	0.34 m	Humerus Radius[a]	0.0105 m
Radius Length[a]	0.255 m	Radius Radius[a]	0.0105 m
Ulna Length[a]	0.255 m	Ulna Radius[a]	0.0055 m
Poisson Ratio	0.5		

[a]Dimensions are calculated from the table of human anthropometry for a 1.8 m height individual (*1*).

Supplementary Note 2.1 — Isometric static testing: force–length characteristics

A static muscle force output can be decomposed into two components—active (F_{act}) and passive (F_{pass}, due to pure visco-elastic mechanics) forces—which in general depend on muscle elongation ($\eta = l/l_0$, where l and l_0 are the current and initial muscle lengths). We first model the quasi-static force output (only function of η) based on experimental isometric data (Main Fig. 1c). In the isometric test, force output is measured when the muscle performs a maximum voluntary contraction (MVC) with its length held constant. As illustrated in Main Fig. 1c, the maximum output force ($F_{\text{max}} = \sigma_{\text{max}} A$, where σ_{max} denotes maximum intrinsic contractile stresses and A the muscle cross-section at rest) occurs for a non-elongated muscle ($\eta = 1$). From Main Fig. 1c we see that $f_{\text{act}}(\eta) = F_{\text{act}}/F_{\text{max}}$ decreases with shortening or extension. For $\eta \leq 1$, the overall muscle force consists of only F_{act} since the parallel elastic component of the membrane wrapped around the muscle fibers is in a slack mode and tension only begins to grow non-linearly for $\eta > 1$ (*1*). We model this parallel elastic mechanism by constructing a piecewise continuous Young's modulus function $E_{\text{membrane}}(\eta)$ of the filaments that reflects this behavior. The two functions $f_{\text{act}}(\eta)$ and $E_{\text{membrane}}(\eta)$ are then empirically determined via a least-squares fourth order polynomial fit of the *in vivo* measurements (*14*) of Main Fig. 1c. The muscle's quasi-static force output is thus expressed as $F_{\text{qs}}(\eta) = F_{\text{act}} + F_{\text{pass}}$, where the passive elastic response F_{pass} directly depends on $E_{\text{membrane}}(\eta)$ (details can be found in Supplementary Note 7).

We then implement these functions into a complete muscle–tendons unit and, to confirm our model behavior against physiological data, replicate *in silico* the isometric test of (*14*). In order to perform the test *in silico*, we initialize our simulated muscle at rest length ($\eta = 1$) and prescribe different external forces F_{set} (inset of Main Fig. 1c). The simulation then evolves the biomechanical system dynamically from $\eta = 1$ to its equilibrium state (i.e. until the muscle no longer changes in length). The output of the simulation—final equilibrium length of the muscle—is then measured. We note that for measurements of cases with $\eta > 1$, we numerically prescribe an additional stretching force on top of F_{set} so that the muscle initially elongates. We then smoothly decrease the additional force to zero, thereby allowing the system to reach its equilibrium state such that $F_{\text{set}} = F_{\text{qs}}$, at which we measure the final length. We can then reconstruct the overall muscle Force-Length curve, which provides a good approximation to the experimental muscle behavior (Main Fig. 1c). This static characterization provides the basis for the more challenging dynamic test in the following.

Supplementary Note 2.2 — Isokinetic dynamic testing: force–velocity characteristics

In a dynamic setting, muscle viscosity causes the velocity-dependent force output to be smaller than its isometric counterpart during length-changing actuation. Isokinetic exercises, which require muscles to perform MVC at constant speed, characterize this behavior. In the experi-

ment conducted in (*15*), a human subject performs maximal isokinetic concentric elbow flexions pushing on a mechanical device that limits the joint angular velocity to a constant, preset value, and muscle torques are measured by an isokinetic ergometer at the joint angle of $60°$ (corresponding to a $\eta = 0.85$ contraction, Main Fig. 1b). From this, given the joint geometry, muscle contraction forces can be estimated. Then, as illustrated in Main Fig. 1d, by varying the angular velocity the bicep dynamic output can be determined. In the figure, six different exercises are reported, each one of them performed twice, once in the morning and once in the evening, thus providing a variational range (*15*).

In our simulations we characterize the damping effects due to muscular viscosity by specifying the internal dissipation rate of the filament. The internal dissipation is modeled as damping forces acting along rods $F_{\text{d}} = \zeta\dot{\epsilon}$ where $\dot{\epsilon}$ is the strain rate, and $\zeta(v)$ is a damping coefficient, so that the dynamic muscle output reads $F_{\text{m}} = F_{\text{qs}} - F_{\text{d}}$. The maximum isometric force F_{max} (and contractile stress σ_{max}, corresponding to the peak of the curve in Main Fig. 1c) is determined from the isokinetic test at zero angular velocity—effectively a static test ($F_{\text{max}} = F_{\text{qs}}(0.85)/f_{\text{act}}(0.85)$). We then consider six simulation cases in which the radius and ulna angular velocities are constrained (via Neumann boundary conditions at their extrema) to replicate *in silico* the experimental setup of Main Fig. 1d. The unknown values of ζ are then empirically determined to match simulated (a complete isokinetic flexion is illustrated in Main Fig. 1b) and measured torque outputs. Finally, to confirm that the obtained ζ are physiologically meaningful, we compare with theoretical estimates (*16*) derived for the Hill model (derivation presented in Supplementary Note 7) and observe that the estimated ζ determined from our simulations is consistent with theoretical calculations (Main Fig. 1d).

In summary, we virtually reconstructed a 3D replica of a human elbow joint and, by taking advantage of isometric and isokinetic tests, modeled individual muscle units actuation so as to realistically reproduce the dynamic and morphological behavior of this system. This calibration/validation study exemplifies our approach to modeling living muscle actuation and serves as a basis for a variety of potential biological and engineering investigations.

Supplementary Note 3 — Hydrodynamic loads via slender body theory

When a body (i.e. swimmer) operates in a flow regime characterized by small Reynolds number (i.e. $Re \ll 1$), we capture the hydrodynamic forces per unit length ($\mathbf{f}_{\text{H}}$) via slender body theory (*17*) as

$$\mathbf{f}_{\text{H}} = -\frac{4\pi\mu}{\ln(L/r)}\left(\mathbf{I} - \frac{1}{2}\mathbf{t}^{\text{T}}\mathbf{t}\right)\mathbf{v}, \tag{1}$$

where μ denotes the dynamic viscosity of the fluid, L and r are the length and radius of the swimmer, $\mathbf{v}$ and $\mathbf{t}$ denote respectively the local velocity and the tangent vector along the body, and $\mathbf{I}$ represents the identity tensor.

Supplementary Note 4 — Bottom-up approach: optimizing increasingly complex architectures.

The section in the main text relative to limbless locomotion in this work aims at illustrating how our model (combined with an optimization procedure) can help to synthesize biological design principles into an engineered device. As such, here we are not so much concerned with the full replica of a real animal (that is the objective of the wing section) or in identifying what exact muscles are critical to a certain gait. Rather, we are interested in distilling general architectural principles or motifs that can be broadly applied in engineering. Because of the emphasis on generally applicable design principles instead of biological detail, we adopt a species-agnostic, bottom-up approach which relies on systematically introducing complexity to a minimal system (as opposed to removal from a complete system in a top-down approach (*18*)). In the following, we show how the progressive additions of muscle groups affects the snake gait. We emphasize here the subtle, yet critical role of optimization: indeed, this allows us to fairly compare progressively more complex models, by identifying their best performance.

As mentioned in the main text, we hypothesize that smooth and fast forward slithering motion can be obtained by a small number of overlapping actuators coupled to a flexible backbone. Following that, we first establish a benchmark for comparison. Independent of snakes' internal anatomy, we know from experimental observations that snakes are able to achieve nearly continuous actuation. Then, if we are not interested in how muscle arrangements lead to this, a simple way to model a snake is to consider it as a slender elastic body actuated via a continuous torque planar wave. Targeting fast gaits, the shape of this function (the torque wave) can be determined via CMA-ES to maximize speed. The obtained torque function is the result of thousands of simulations guided by an evolution scheme until no further improvement in forward speed is observed. This continuum model, despite its simplicity, represents the upper bound in terms of attainable speed and gait smoothness: indeed, from a mathematical perspective, the obtained torque profile is a $\mathcal{C}^\infty$ function, i.e. a function infinitely smooth and differentiable, and as such, it allows for the greatest level of control (torque prescribed at each infinitesimal cross section). Biological snakes clearly approach this limit. Thus, the optimized continuum snake establishes a computational reference, but it does not tell us anything about how muscular arrangements work. The goal is then to generate increasingly complex muscular snakes, and test whether they can approach the reference's speed and kinematics, and whether muscle overlap naturally arises as a critical architectural feature.

We start by considering the simplest possible muscular architecture in which only one muscle–tendon pair (i.e. two equal antagonistic muscle–tendon groups) is included in the snake model. Depending on where the pair is located along the body, its span and actuation, different gaits can be achieved. Then, we ask CMA-ES to identify these parameters so that the one-muscle snake can move as fast as possible. The solution found is illustrated in the first column of Supplementary Fig. 2. As immediately noticeable, and expected, this architecture is simply incapable of reproducing the reference gait and speed.

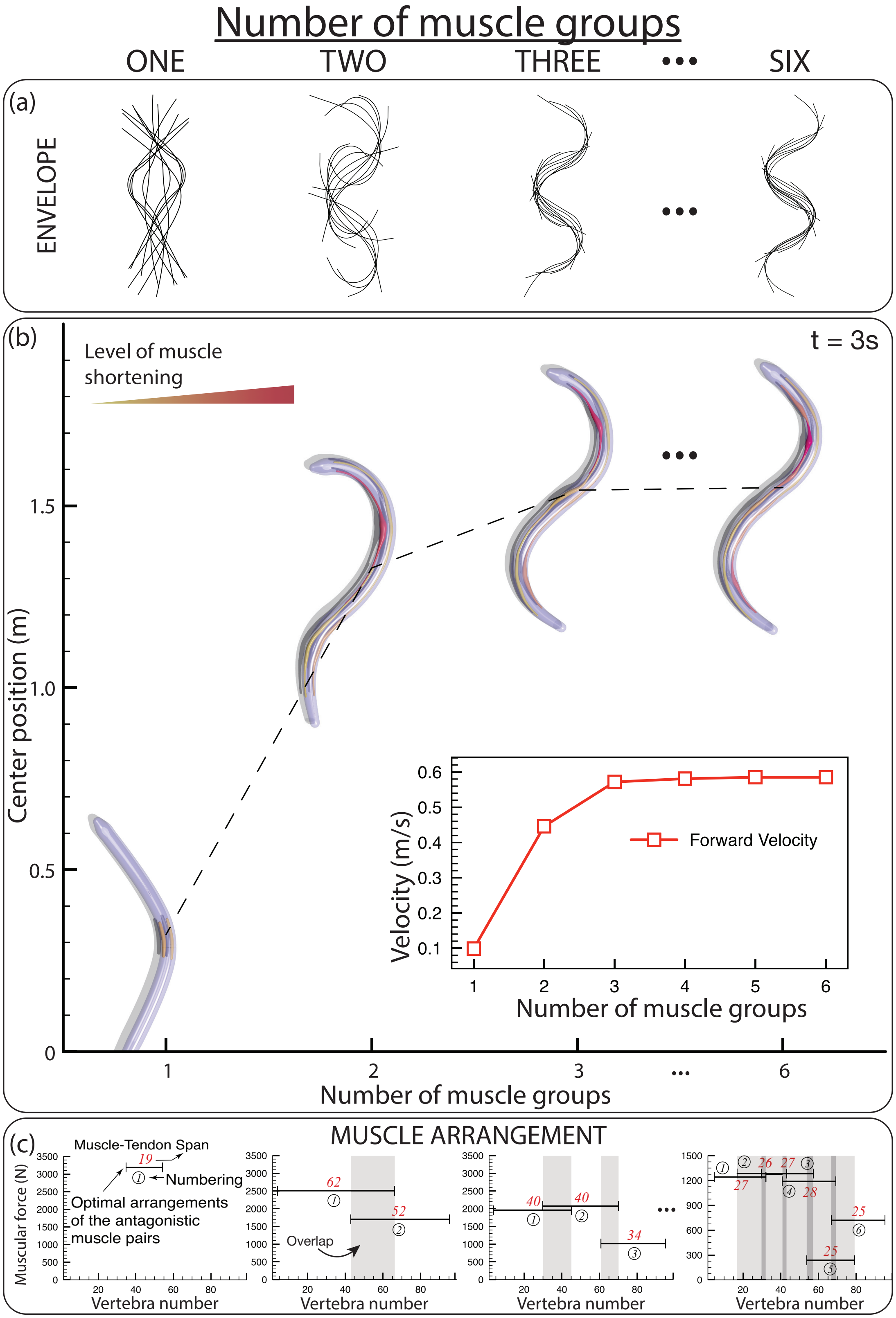

Supplementary Figure 2. Optimized designs for snakes equipped with different number of muscle pairs (increasing

from left to right) detailing the **(a)** gait envelopes, **(b)** center-of-mass positions at t = 3s and the corresponding average forward velocities (inset), and **(c)** optimal muscle arrangements and span across vertebrae as well as the peak contraction forces of these muscles.

Next, we add complexity by introducing a second muscle pair. Again, we let the optimizer determine the two-muscle architecture muscular layout and actuation parameters, so as to maximize forward speed. As can be seen in the second column of Supplementary Fig. 2, the snake's speed improves and the gait starts resembling the reference's one. We also note that the optimizer finds on its own a solution in which muscles overlap (in Supplementary Fig. 2c each line in the plot represents a muscle–tendon group pair: the line indicates the span and location across vertebrae (x-axis), while its y-position is the peak muscle force). We refer readers to the exploded rendering based on a four-muscle model in Supplementary Fig. 3c for better visual intuition. We note that muscle-overlapping is not built into the model nor the optimization. The optimizer spanned candidate solutions with both overlapping and non-overlapping muscles and found the former design to be most effective in terms of locomotion speed. This procedure highlights the importance of using an optimization method to fairly compare different architectures according to the same standards: indeed, within the class of one-muscle and two-muscle architectures, we identified the configurations that produce the fastest gait, and compared those.

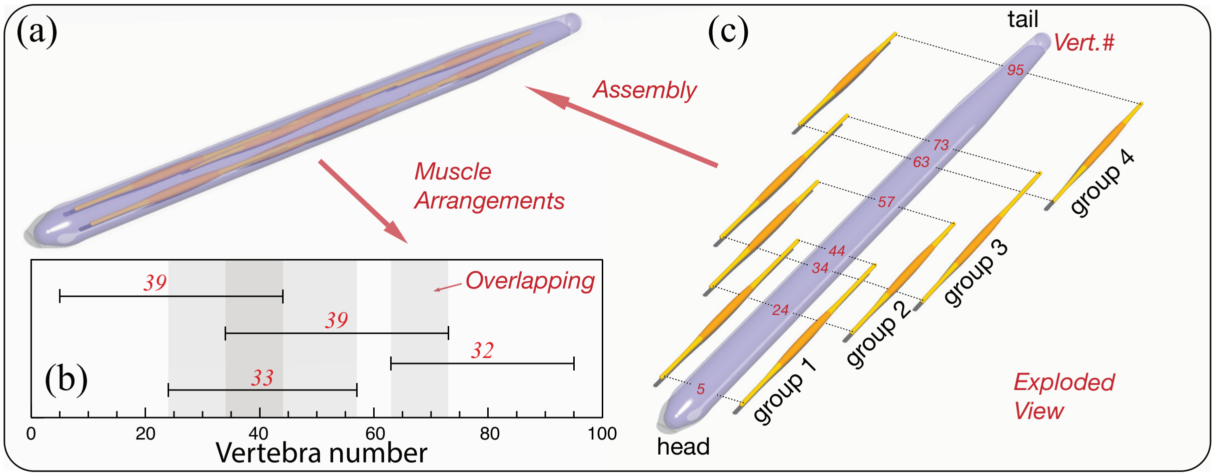

Supplementary Figure 3. **(a)** Full assembly of a four-muscle snake which exhibits characteristics of **(b)** overlapping arrangements in the muscle–tendon groups that each span 30-40 vertebrae. **(c)** An exploded view of the snake's muscular architecture illustrates the muscle–tendon pairs' position relative to the snake's skeleton, providing visual clarity of the overlapping arrangement.

We then keep increasing the number of muscles to three, four, five and so on to see how the architecture evolves together with the associated speed and gait kinematics. Supplementary Fig. 2 illustrates this process. As can be seen, as the number of muscles increases, the solutions identified by CMA-ES tend to approach the speed and gait of the reference one, as well as closely resemble experimentally observed gaits (Fig. 3e in the main text). The effect of adding muscles becomes less and less conspicuous, and plateaus after four muscles, hence our choice in the demonstration presented in the main text. Supplementary Fig. 3 illustrates an exploded

view of the four-muscle snake architecture (the same snake illustrated in Fig. 3g in the main text), providing visual clarity in the overlapping of muscles–tendon groups.

The procedure above shows that indeed a few simple actuators can produce fast, smooth gaits, and muscle overlapping naturally emerges as a favorable solution. Indeed, by scanning through all generated solutions, we observe that non-overlapping arrangements consistently underperform overlapping ones, with top speed degradation ranging from $\sim$25% to $\sim$60%.

Supplementary Note 5 — Numerical modeling of feathered wing

Supplementary Note 5.1 — Modeling of barbs in a feathered wing

In the feathered wing example in the main text, we model the geometrical and material properties of the rachis so that its bending stiffness corresponds to those found in nature (*19*). This is difficult to do in the case of barbs. Indeed, the number of barbs differ significantly between species and between feathers within a species (*20*). Moreover, barbs interlock with one another to increase their effective stiffness (*21*). In view of all these difficulties, we adopt an engineering approximation and lump 5 biological barbs into a single computational one . The properties of our computational barb are chosen so as to conserve the wing area (*22*) and the estimated aggregate bending stiffness (chosen to be 5 times larger than the numbers reported in (*21*)). The geometrical and mechanical properties of the feathered wing example are given in Supplementary Table 2.

Supplementary Table 2. Geometrical and mechanical properties of the feathered wing.

**Parameters**	**Values**	**Parameters**	**Values**
Number of feathers	19	Feather length	$8.4 - 18$ cm
Rachis density	0.0025 g cm^{-3}	Rachis radius	1 mm
Rachis bending stiffness (EI)	5.04 N $\cdot$ mm^2	Barb density	0.0025 g cm^{-3}
Barb radius	0.6 mm	Barb bending stiffness (EI)	0.651 N $\cdot$ mm^2
Number of computational barbs	200	Total wing area	650 cm^2
Poisson Ratio	0.5		

Supplementary Note 5.2 — Approximation of aerodynamic loads on a feathered wing

We approximate the effect of the environment on the wing structure via a reduced order model in which the drag for $\mathbf{F}_\text{D}$ scales quadratically with the local body velocity as

$$\mathbf{F}_\text{D} = -\left(\frac{1}{2}\rho|\mathbf{v}|^2 C_\text{d} A\right) \cdot \frac{\mathbf{v}}{|\mathbf{v}|} \tag{2}$$

where ρ is the air density, $\mathbf{v}$ is the local body velocity, $C_{\mathrm{d}} = 1.2$ denotes the drag coefficient (as determined in (*23*) for smooth cylinders) and A is the cross-sectional area of each filament that forms the feather. We also note that the drag coefficient remains fairly constant in the Reynolds regime (500–10000) relevant to takeoff flight mode of the wings. We are fully aware that this simple model cannot capture the complex unsteady aerodynamics associated with flapping flight. Nonetheless, it provides a preliminary estimate. We underscore the qualitative character of this specific demonstration.

Supplementary Note 5.3 — Modeling assumptions

The full-scale modeling of the feathered wing is a numerically challenging and demanding problem, as it consists of a complex interconnect between multiple, disparate components. All these components dynamically interact with each other and the environment, across multiple length and time scales. Moreover, biological wings entail a number of components that are not included in our simulations such as, for example, flesh, skin, connective tissues and secondary muscles. In addition to that, during wing flapping, birds can leverage feedback control strategies so as to dampen out undesired mechanical modes that may arise. To qualitatively account for some of these effects, our wing model relies on the following set of modeling assumptions.

- Effect of gravity. We ignore the effect of gravity in the wing simulations. This assumption follows from the absence of most of the dense biological components (such as flesh, connective tissues, etc.) in our computational model. These components are estimated to have the largest effect on the system outcome due to gravity. Furthermore, gravity represents a constant force offset throughout the duration of the simulation. Hence we expect it, at the leading order, to only quantitatively shift the forces exerted by our muscles without changing the character of the kinematics of the wings.

- Joints in the bone–rachis–barb assembly. Our wing model assembles multiple, disparate components—ranging from stiff, low-mass and low-density barbs to soft, highly flexible muscles—into a single biologically aggregated entity. Owing to the wide range of time-scales inherent in such dissimilar components, our numerical system demonstrates stiffness in temporal integration, constraining the allowable (stable) time-step to unfeasibly small values ($\sim 10^{-10}$ s). Upon further investigation, we identify that the rachis–barb assembly contributes predominantly to this numerical stiffness, due to their high Young's modulus, low density, thin geometry and high accelerations they are subject to.

  To overcome these numerical limitations, for the rachis–bone connections, we employ a modified version of the fixed joint presented in the main text. In this variant, the penalty forces that enforce physical connections are computed as in the fixed joint case, so that loads are exchanged among the rods. However, feedback torques (rachis to bone) are not calculated, but rachis's orientation is constrained to move kinematically with the bone. This is achieved computationally by imposing the frames of the rachis' connecting element to match the orientation of the connected element of the bone. Adopting such a

joint effectively filters out fast time-scales associated with the rachis in our system, as we discount torque calculations while computing and transferring forces. The choice of such a joint is not arbitrary, but is biologically motivated by the fact that remiges (primary and secondary feathers) are anchored to the bones through stiff ligaments, and are hence forced to effectively move kinematically.

The rachis–barbs connections are the numerically stiffest components in our model due to their extreme light weight and thin structure combined with their large Young's modulus and the high accelerations that they experience (the highest in the entire system). We then take a similar approach as above. Barbs connection orientation is kinematically constrained to the rachis, and force loads are transferred from the rachis to the barbs. The response force load from the barbs to the rachis is then computed as a linearly decaying (along the rachis from base to tip) sum and directly transferred at the base of the rachis to the bone. The linear decay has been modeled based on the minimal study of Supplementary Fig. 4, which shows how loads applied at different locations along the rachis are "felt" less and less at the base of the rachis itself as they are applied closer and closer to the tip of the beam. This is only true in a dynamic setting and for the time scales characteristic of wing actuation. In a static setting, the load at the base would be of course the direct sum of all forces applied along the beam.

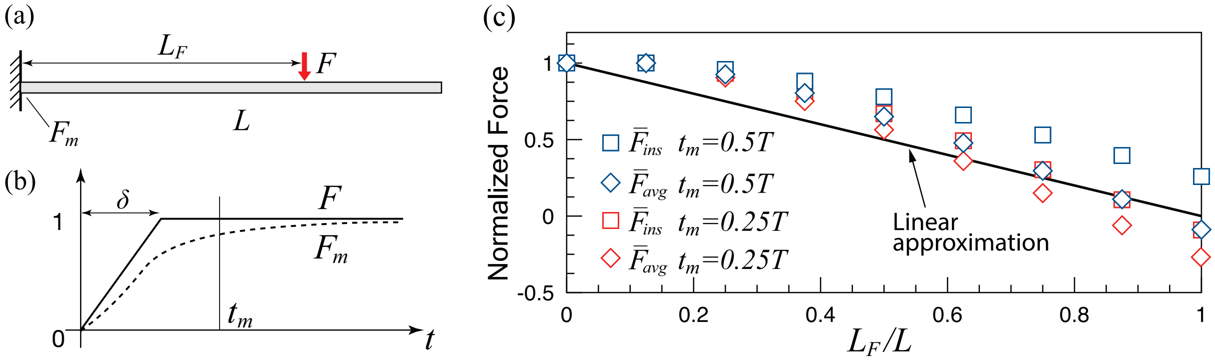

Supplementary Figure 4. **(a)** A minimal cantilever-beam setup of a rachis of length L connected to the bone (wall). We mimic the aerodynamic forces of a barb using a transient point force F at some length L_{F} and measure the reaction forces F_{m} at the wall. This force F is **(b)** ramped linearly from 0 to 1 (non-dimensionalized) within some time δ chosen here to be $0.5t_m$, where t_m corresponds to the time of measurement. This choice of δ realistically models the time-scale of barb motion, being less than $0.25T$ (corresponding to maximum wing-loading/displacement) while being much greater than the elastic-wave time-scales($0.001T$). We record the induced reaction forces at some time t_{m}, which **(c)** varies between $0.25T$ to $0.5T$ (which approximately correspond to the time scales that dominate the bone joint angles dynamics). These non-dimensional induced forces are then reported against varying barb position L_{F}/L at these two extreme instants, depicted as red ($t_{\mathrm{m}} = 0.25T$) and blue ($t_{\mathrm{m}} = 0.5T$) symbols in this figure. Squares indicate instantaneous force measurements (non-dimensionalised against peak $F = 1$) while diamonds indicate average force measurement from $t = 0$ to $t = t_{\mathrm{m}}$ (non-dimensionalised against the area under $F - t$ curve). The force response demonstrates a near-linear decay with increasing barb length, which we then feed into our full-scale wing model.

Numerically, the above described connections relax the fastest time scales, rendering our simulations feasible. Moreover, they allow to account for force loads experienced by

the feathers and their impact on the wing bone structure and dynamics. On the other hand, physically, they also have the effect of filtering out some of the associated torques. The justification of this approximation stems from the nature of aerodynamic forces and the structure of the computational wing. A bone–rachis–barb system is constrained to move only in one plane due to the elbow joint connection. The torques generated by the aerodynamic loads predominantly act out of this plane—such torques are balanced out internally by the elbow joint. Hence the dynamics of the bone–rachis–barb system is itself not significantly affected whether or not such torques are accounted for. The elbow joint in turn propagates all such out-of-plane torques as a torsional component to the spherical shoulder joint. Thus, this effect is not accounted for. Nonetheless, (1) these torques are a second-order effect relative to the ones generated by muscle contractions, and (2) in avians the shoulder-humerus connection is not perfectly spherical, and rotations are attenuated and additionally constrained by the surrounding connective tissue. Moreover, in real birds, muscle contraction adjustments are executed via feedback control so as to correct for torque fluctuation.

Therefore, the above approximations prevent numerical stiffness in our wing setup, rendering simulations possible, while still capturing the most salient traits of the underlying biophysical behavior. Moreover, if one chooses to avoid such approximation and fully resolve the temporal scales computationally, several potential numerical approaches exist. Implicit time integration schemes and local time-stepping (LTS)/multi-rate time-stepping schemes are two such techniques that can be employed to counter stiffness issues. Their feasibility, efficiency and accuracy in integrating the dynamics of assembled Cosserat rods is an area of active research that we are currently pursuing.

Supplementary Note 5.4 — Close-up visualization of feathered wing

In order to provide enhanced clarity in visualizing the wing muscular arrangement, we present here a close-up view from multiple perspectives in Supplementary Fig. 5.

Supplementary Note 6 — Impact of twist and shear modes and potential opportunities

Here we highlight the importance of capturing twist and shear modes in complex systems. While the role of twist and/or shear can be easily predicted *a priori* for simple problems (e.g. axial stretching of an elastic rod does not introduce any twist or shear), their significance in more complex architectures comprising multiple elements (i.e. different geometries, mechanical properties, orientation, etc.) interacting non-linearly among one another and with uncertain environments (i.e. uneven terrain, turbulent flows, etc.) are often less intuitive.

In each of the following demonstrations, we perform numerical twist-hardening, shear-hardening, and both twist- and shear-hardening experiments, wherein we adjust the material

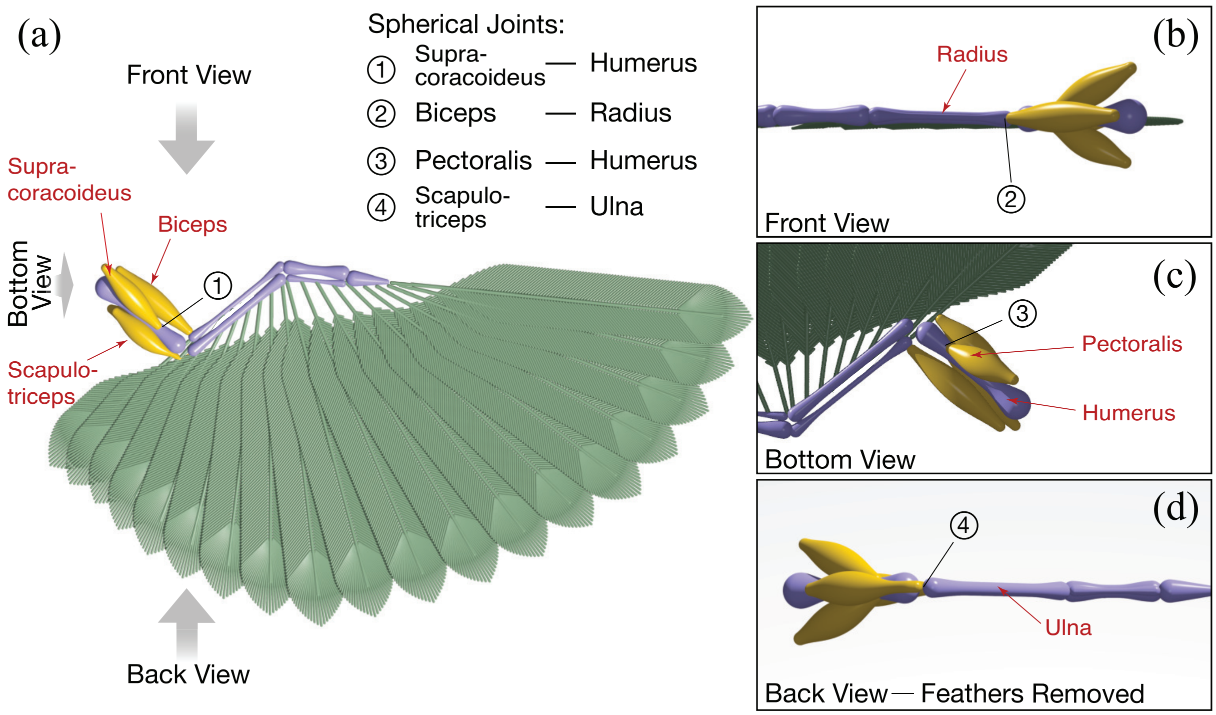

Supplementary Figure 5. **(a)** The full assembly of a feathered wing composed of bones (humerus, ulna, radius—purple), muscles (supra-coracoideus, biceps, pectoralis, scapulo-triceps—yellow) and feathers (green). A close-up view of the wing from the **(b)** front, **(c)** bottom and **(d)** back view (with feathers removed to avoid visual occlusion).

properties affecting shear and twist accordingly. Then, we record system outputs and compare them with the original result (no artificial hardening) to highlight the individual and simultaneous effects of twist and shear. In some instances, we also comment on the effects of softening twist and shear. We perform these studies on the architectures discussed in the main text (elbow joint, swimming robot, walking robot, soft snake, and feathered wings). Moreover, two additional demonstrations are introduced to highlight the potential opportunities relative to the deliberate use or modulation of twist and shear specifically.

Supplementary Note 6.1 — The human elbow joint

We first consider the human elbow joint (Supplementary Fig. 6a) and test the individual and simultaneous effects of twist- and shear-hardening. Since the bones are mainly stiff structures, we only perform twist- and shear-hardening for the muscle and tendons. We simulate the elbow flexion (Supplementary Fig. 6a) and compare the time-varying joint angle for different hardening configurations in Supplementary Fig. 6b. As can be seen, neither individual nor simultaneous hardening of twist and shear affect the dynamic system outcome. This is not surprising given that the muscle contractions only act axially along the biceps brachii, and the elbow motion is constrained in the plane identified by the bone structure. Therefore, the problem retains

its original nature, and no twist and/or shear deformation modes are introduced. These tests help us rule out the potential introduction of numerical artifacts: indeed shear and twist stiffnesses are artificially increased by several orders of magnitudes and no spurious oscillations or instabilities arise.

In the next study case, we illustrate instead how the use of twisting modes may represent an opportunity. Twist in fact is modulated in the form of artificial muscles, that can be used as assistive devices to recover normal joint flexion when the muscles are weakened or injured.

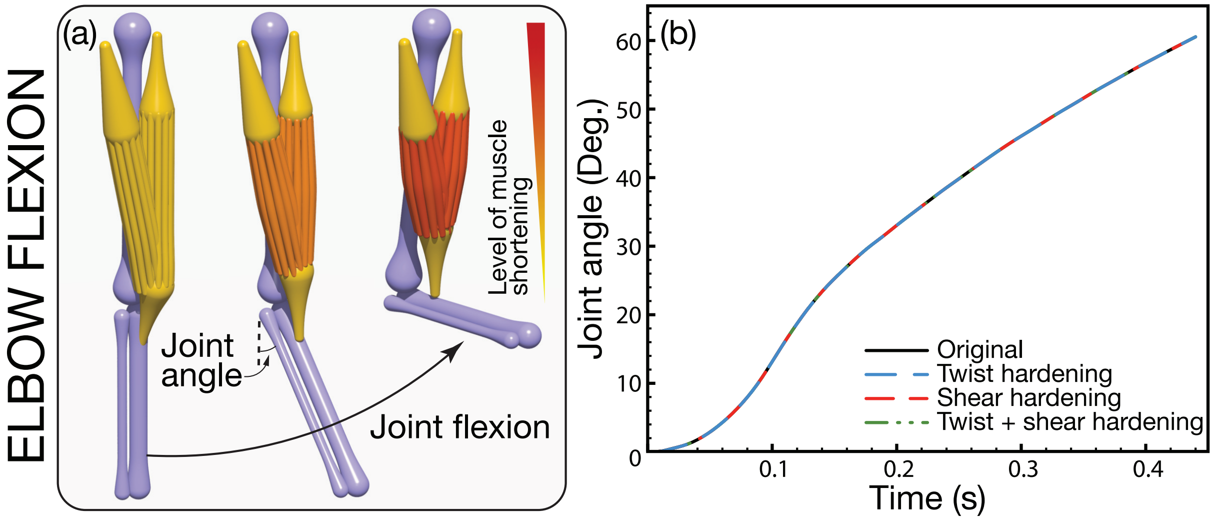

Supplementary Figure 6. **(a)** The full assembly of an elbow composed of bones (humerus, ulna and radius—purple), muscles and tendons (biceps brachii—yellow/red). The original muscles ($E = 10$ kPa, $G = 2E/3$) are hardened for twist and shear by a factor of 10000 (i.e. $10000G$). The original tendons ($E = 500$ MPa, $G = 2E/3$) are hardened for twist and shear modes by a factor of 10 (i.e. $10G$). **(b)** Resulting time evolution of joint angle for hardening twist (blue), shear (red), and both twist and shear (green) are compared with the original elbow performance.

Supplementary Note 6.2 — Rehabilitation using artificial, assistive muscles

Here we put into context the role of twist in complex architectures relative to our well-validated elbow joint case via the assimilation of twist-based artificial muscles. This demonstration also serves to further highlight the robustness and versatility of our modeling approach and showcase its ability to address patient-specific kinesiological needs. We consider the following setting of human arm rehabilitation, wherein we integrate biological and artificial components. We replicate the human arm-elbow setup (Main Fig. 1a), and mimic an injury/fatigue to the biceps by impairing its constriction (modeled numerically by rendering some fibers passive). To regain functionality of the arm, we enhance the impaired biological muscle with an external artificial muscle (shown in Supplementary Fig. 7a below), modeled as a soft (i.e. stretchable), highly twisted Cosserat filament. This device can generate longitudinal contractile forces by releasing stored twist into stretch and is inspired by the mechanical response shown be highly-coiled

fishing lines of (*24, 25*). The twist release in our simulations is achieved using the same mechanism as (*26*) (which mimics the experimental release mechanism of running a thin electric wire along the soft coiled element: upon heating, the radius of the coil expands, releasing twist which in turn is converted to contraction), shown in Supplementary Fig. 7d. We refer the reader to (*26*) for more details on the underlying physical mechanism and numerical implementation details, and note that the kinematic and material parameters used in this study are detailed in Supplementary Table 3.

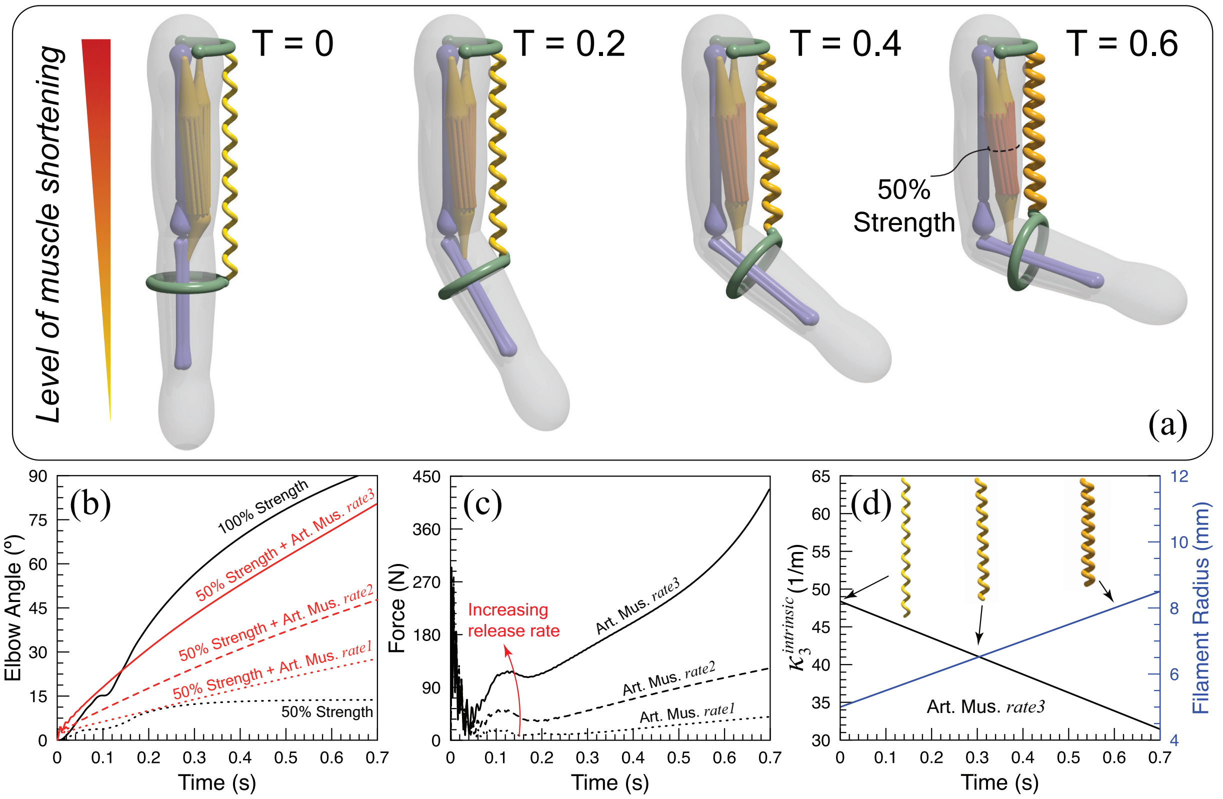

Supplementary Figure 7. Rehabilitation of a human elbow: **(a)** While an injury to our elbow joint setup impairs its ability to generate forces and hence lift a weight, it can be compensated for by applying external artificial muscles which provides restoring forces by releasing internal twist. The magnitude of these restoring forces is modulated by the rate of twist release. We show this by plotting the **(b)** elbow angles and **(c)** artificial muscle forces for three different release rates (dotted, dashed and solid lines). For reference, the healthy (100% strength) and injured (50% strength) cases are also depicted in the former. **(d)** Shows the release of twist (black lines, represented by internal curvature k_3), achieved by increasing the filament radius (blue lines) for the case of highest twist release. (Inset) shows the structure of the artificial muscle at different instants.

As the artificial muscle shortens, it effects an end force over the contractile time interval. The magnitude of this force can be modulated by the rate of twist release—more force is generated the faster we release twist. This is shown in Supplementary Fig. 7c, with twist release rate (and hence forces) increasing in the direction of arrows. We leverage these contractile forces for enhancing the functionality of an injured/fatigued arm, for cases where it fails to perform satisfactorily. One such scenario is shown visually in Supplementary Fig. 7a. A healthy elbow

Supplementary Table 3. Geometrical and mechanical properties of the Artificial Muscle (AM)[a].

**Parameters**	**Values**	**Parameters**	**Values**
AM E	30 GPa	AM density	1.67 g cm^{-3}
AM initial filament radius a	0.005 m	AM helix radius r	0.01 m
AM initial core length L	0.425 m	AM initial number of turns n	9
Poisson Ratio	0.5		

[a] Muscle, Tendon and Bone parameters are retained from Supplementary Table 1.

is shown to be able to lift a weight placed at the end of the radius, while an injured one (50% less contractile stresses) cannot do so. To quantitatively measure muscle performance in this case, we track the elbow angle with time, with the healthy muscle attaining higher elbow angles than the injured one as shown in Supplementary Fig. 7b. Upon enhancement with artificial muscles, and by modulating the rate of twist release, we can obtain desired lifting performance so as to approximately match the patient's needs.

This demonstration of rehabilitation underscores the importance of capturing bend, stretch, twist and shear physics in a general setting, and the ability of our numerical approach to do so, thereby addressing patient-specific needs.

Supplementary Note 6.3 — Cell-powered swimming flagella robot

Here we consider the bio-hybrid cell-powered swimming flagella robot of (*27*) and model a one-to-one computational replica (Supplementary Fig. 8a) with the same properties as those used in the main text. Then, we perform twist- and shear-hardening for the entire structure (cell and substrate) and compare the forward displacement of the swimmer's center of mass for the different hardening configurations as illustrated in Supplementary Fig. 8. As can be seen, twist- and shear-hardening do not affect the swimmer's performance. This makes physical sense for several reasons. Firstly, the particularly thin structure of the swimmer is barely susceptible to any shearing effects to start with. Indeed, shear becomes less and less relevant for thinner and thinner beams. Secondly, the cell-powered actuation produces a highly-localized torque which causes only in-plane bending, and no element in the structure or in the (very viscous) fluid environment breaks the 2D symmetry of the system to excite any 3D motion that may lead to twisting or shearing. Thirdly, the swimmer operates in the Stokes flow regime ($Re \sim 10^{-2}$), a viscous environment which dampens all deformation modes rather than exciting them.

Supplementary Note 6.4 — Bio-hybrid walking robot

Here we consider the walker design of (*28*) with twist- and shear-hardening of the entire structure (muscle and scaffold) and compare walking performances. As can be seen in Supplementary Fig. 9, both twist- and shear- hardening significantly affect the walker's forward displacement, with speed varying up to $\sim$43% between the fastest (shear-hardened) and the slowest (twist-hardened) walker. We relate this to the slip-and-stick nature of surface friction and con-

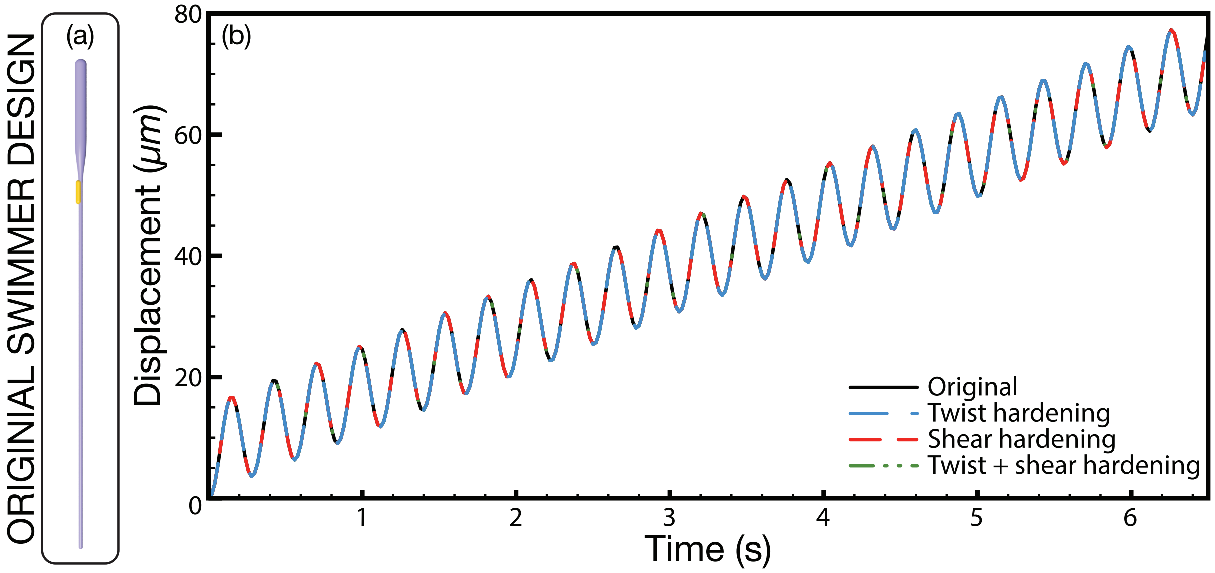

Supplementary Figure 8. **(a)** One-to-one computational replica of cell-powered swimmer of (*27*). Both the original contractile cell (yellow, $E = 30$ kPa, $G = 2E/3$) and the substrate (purple, $E = 3.86$ MPa, $G = 2E/3$) are hardened for twist and shear by a factor of 100 (i.e. $100G$). **(b)** Resulting time evolution of the swimmer's center position for hardening twist (blue), shear (red), and both twist and shear (green) are compared with the original swimmer performance.

tact, which produces three-dimensional, localized, intermittent and impulsive forces. Thus, this environment has the tendency to excite all deformation modes instead of dampening them as in the case of viscous fluids, thereby introducing (or magnifying) the twist and shear modes in the structure. Shear modes in particular (red curve in Supplementary Fig. 9b) are found to play an important role. We know from Timoshenko beam theory that shear causes a beam to deflect more than bending alone. This, translated in our walker setup, causes the local normal forces exerted by the walker on the substrate to be modulated differently in the shearable and unshearable cases, leading to different speeds. This is in agreement with biological (*29*) and robotic (*30*) studies in which friction manipulation is highlighted as a key factor in locomotion performance. For example, snakes lift part of their bodies to modify their grip on the ground.

Then, in the next demonstration, we further exemplify the role of twist and shear in modulating friction in our snake models, showing how these modes can affect performances both qualitatively (fail or success in overcoming terrain features) and quantitatively (different forward speeds and kinematic envelopes).

Supplementary Note 6.5 — Soft, limbless, slithering snakes

We now consider the continuum soft snake model of (*31*), whose muscular actuation is described by a continuous torque function along its body (Supplementary Fig. 10a), and perform twist- and shear-hardening experiments (details in image caption). The snake's center-of-mass position are compared between these different configurations in Supplementary Fig. 10b. The

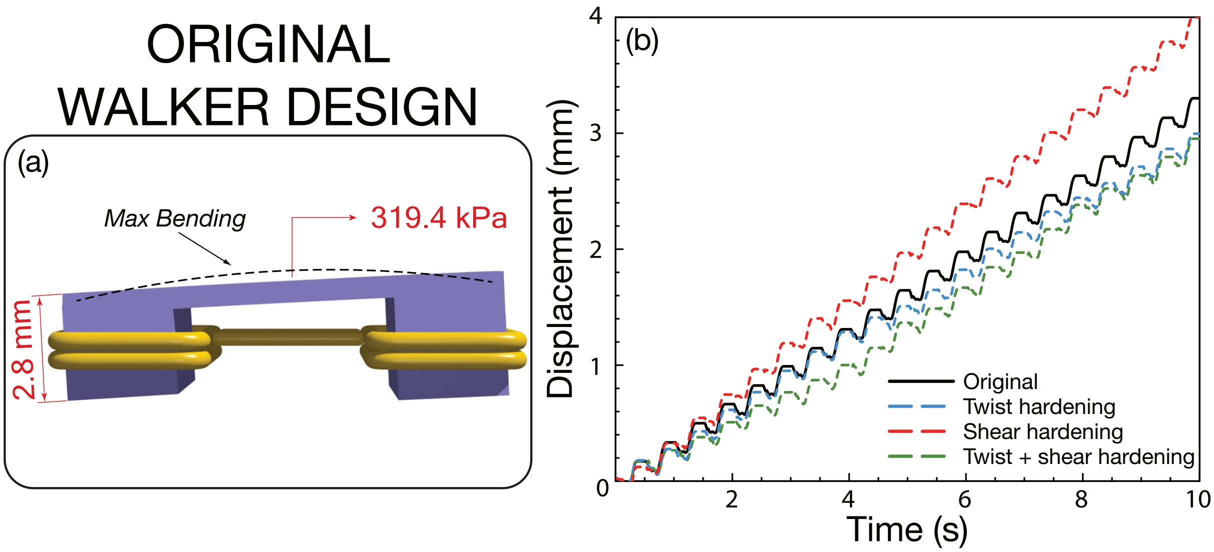

Supplementary Figure 9. **(a)** One-to-one computational replica of bio-hybrid walker of (*28*). The original muscle (yellow, $E = 10$ kPa, $G = 2E/3$) is hardened for twist and shear modes by a factor of 10 (i.e. $10G$). The original scaffold (purple, $E = 319.4$ kPa, $G = 2E/3$) is hardened for twist and shear modes by a factor of 10000 (i.e. $10000G$). **(b)** Resulting time evolution of the walker's displacements for hardening twist (blue), shear (red), and both twist and shear (green) are compared with the original swimmer performance.

resulting trajectories (Supplementary Fig. 10b) exemplify that twist and shear indeed affect the undulatory gait, both in terms of forward speed (up to 10% differences) and the lateral gait displacements (Supplementary Fig. 10c) which are found to differ by as much as 60%. These differences in slithering performance again relate to the local normal forces that are modulated differently (as also observed in the walker case above) due to differences in shearability of the snake.

In the next study case, we further highlight the role of twist, and shear in particular, in slithering gaits when the environment is uneven, thereby introducing terrain features. This is perhaps the most striking example on the importance of shear among the proposed set of investigations.

Supplementary Note 6.6 — Snakes slithering on uneven terrain

We further focus on the effects of shear in a slithering snake by casting it in an environment where three-dimensional deformations are important. Indeed, so far we have considered snakes propelling on perfectly flat surfaces. Now we introduce terrain features, so as to give prominence to the out-of-plane (i.e. 3D) shear mode. Then, we perform a proof-of-concept study in which the barrier shown in Supplementary Fig. 11 is considered, thereby challenging the snake to overcome the effect of gravity up the slope. We then consider snakes with and without shear hardening actuated by a continuous torque profile, similar to (*31*). Due to the difference in compliance of these snakes, we expect them to conform to the shape of the substrate differently, thus affecting their contact with the ground. Since limbless locomotion relies crucially on friction

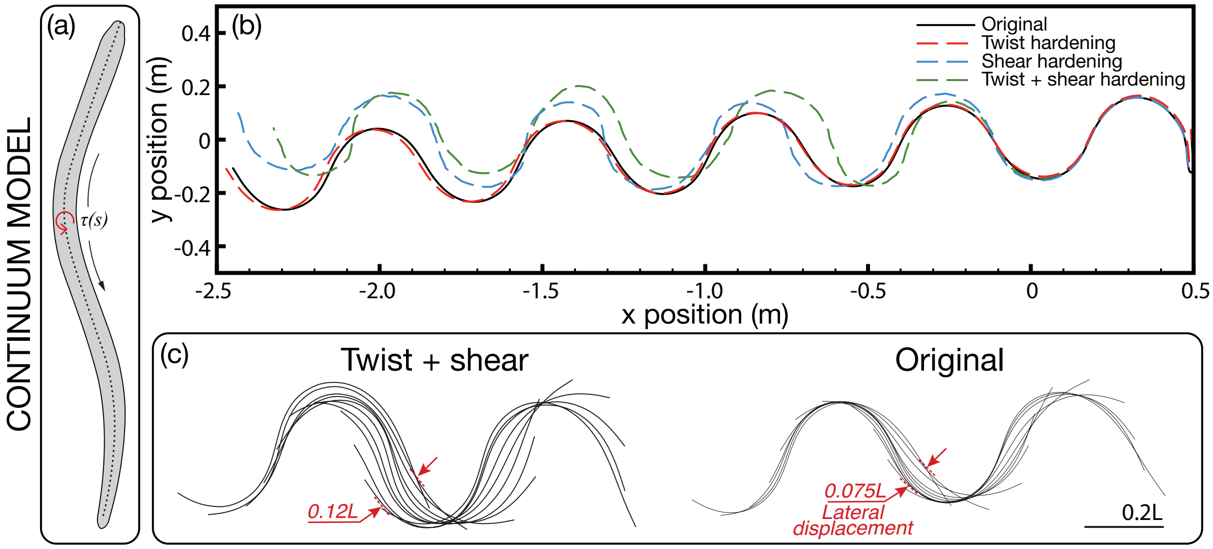

Supplementary Figure 10. **(a)** Continuum soft snake model of (*31*). The original snake's skeleton ($E = 10$ MPa, $G = 2E/3$) is hardened for twist and shear modes by a factor of 25 (i.e. $25G$). **(b)** Resulting center of mass position of the snake for hardening twist (red), shear (blue), and both twist and shear (green) are compared with the original snake's slithering performance. **(c)** Comparison of gaits and lateral displacements between hardened (left) and original (right) slithering snake (Scale bar, 0.2L).

modulation, we hypothesize that these snakes would exhibit different behaviors.

Indeed, when testing these snakes in this environment (Supplementary Fig. 12), we see that the hardened snake (blue) manages to slither up the slope barrier successfully, while the one without hardening (red) cannot. Hence, the presence or absence of shear determines the difference between success and failure. Upon comparing the snake centerline trajectories at different temporal snapshots (Supplementary Fig. 12), we see that shear hardening of the snake (hence, making it less compliant) results in uneven contact with the substrate while slithering up (see Supplementary Fig. 12 at 7.5T). This uneven contact in turn modulates net frictional forces (both propulsive and opposing) for successful locomotion over the barrier. This is again consistent with the biological observations of (*29*) where snakes lift part of their bodies to modulate friction, thus locomotion.

This study then demonstrates dramatic qualitative differences in the system outcomes (success/failure in overcoming barrier) when shear is accounted for. This particular scenario, underscores the risk of designing a robotic snake using for example the Kirchhoff rod model. Indeed, unless specific precautions are taken, soft robots are made of shearable materials (*30*). Thus, the Kirchhoff model would under-predict the snake compliance, hence it would not detect problems in climbing up the slope. In contrast, the actual, shearable robot will struggle and potentially fail. In this specific instance then, the use of Cosserat rods is more appropriate for actual engineering design, as they will be able to detect the problem and inform roboticists on the need to account for it.

We conclude by noting that this proof-of-concept study points to a detrimental effect of shear. Nonetheless, there might be cases in which more shearable materials might help, de-

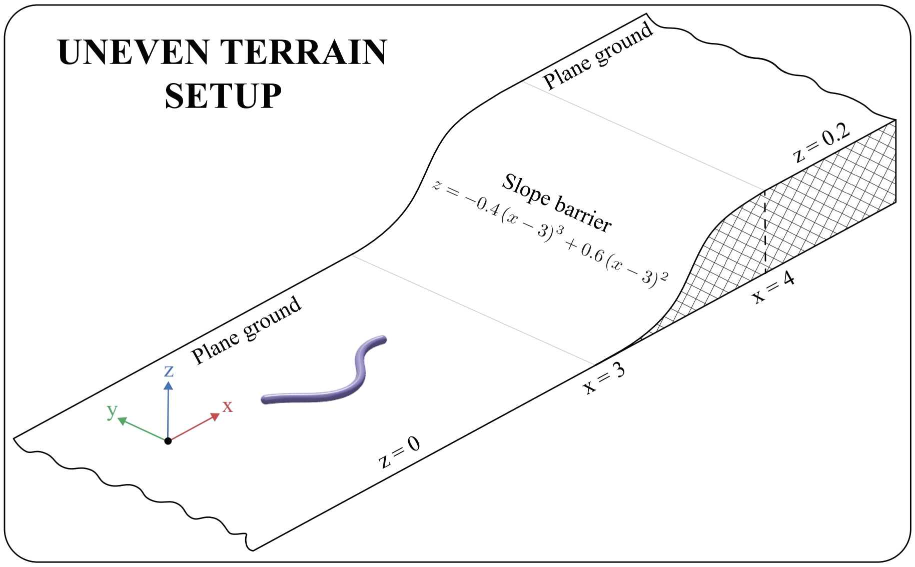

Supplementary Figure 11. Uneven terrain setup (in the $x - z$ plane) demonstrating the effects of shear and twist hardening on the trajectory of a snake actuated with a continuous torque profile. The setup consists of two half-planes at $z = 0$ and $z = 0.2$. These planes are connected by a sloped barrier, which is the polynomial function described by $z = -0.4\,(x-3)^3 + 0.6\,(x-3)^2$ for $3 \le x \le 4$, chosen for $\mathcal{C}^1$ continuity with the half-planes at the line of intersection, while also being symmetric about $x = 3.5$. The barrier is placed at a sufficient distance from the initial position of the snake $(0, 0, 0)$ to account for initial startup transience.

pending on the context and envisioned tasks. In our opinion, this is a telling example, and opens up a new avenue of investigation which we will pursue in the future.

Supplementary Note 6.7 — Feathered wings

Here we consider the wing from the main text, and test the potential impact of twist and shear. We start by hardening twist and shear mode of the muscles, tendons and feathers of the wing and compare output kinematics of the three joints. As can be seen in Supplementary Fig. 13, hardening both twist and shear modes by a factor of 10 quantitatively alters the resulting kinematics of the wing, although not significantly (up to $\sim$3% maximum difference in joint angles). We also note that no change is observed in the kinematics upon further hardening (we tested 25, 50 and 100 times hardening factors).

These small changes were surprising at first, as we expected the complexity of this structure to amplify the role of shear/twist. Nonetheless, the particular geometries and materials of the various elements justify this outcome. Indeed, bones are effectively rigid objects given their high Young's modulus (the highest among all elements), thus further hardening does not change their behavior. Barbs and rachis are made of stiff materials too. Moreover, they are very thin, rendering them less susceptible to shearing modes. A similar argument is valid for tendons as well. Then, the most sensitive elements are the muscles. Nonetheless, the stiff tendons seem to be able to keep them under sufficient tension, so that the effects of shear and twist are contained.

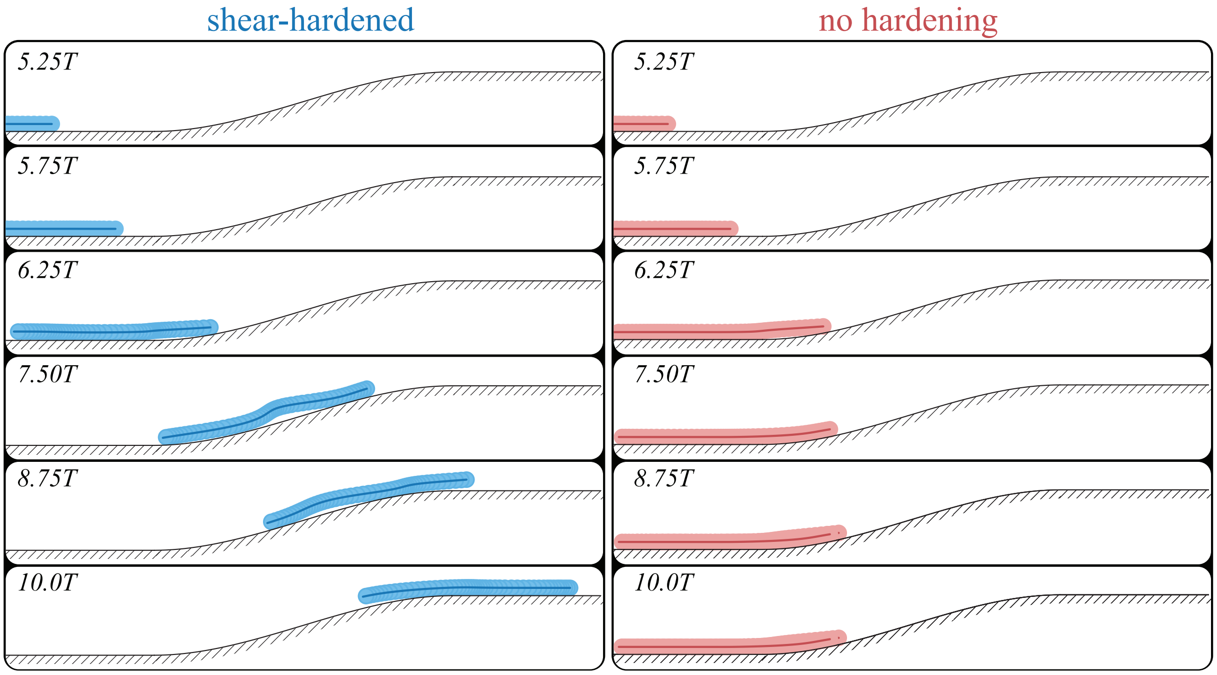

Supplementary Figure 12. Snake locomotion on uneven terrain: We compare the trajectories of an unhardened and shear-hardened snake across the uneven terrain of Supplementary Fig. 11. Hardening the shear and twist by a factor of 100 (i.e. $100 \times G$) leads to different qualitative outcomes. While a shear-hardened snake (left, blue) can overcome the sloped barrier given enough time, the unhardened snake (right, red) simply cannot do so. The snapshots at different times demonstrate how the hardened snake lifts its body off the substrate thus modulating frictional forces to overcome the effect of gravitational forces. The parameters for the non-hardened snake used in this setup are listed below, where the quantities and symbols retain their definition from our main manuscript and Gazzola et. al, 2018. We add that no static or rolling friction is considered in this setting. Settings: length $L = 1$ m, radius $r = 0.025$ m, density $\rho = 10^3$ kg m^{-3}, $T_{\mathrm{m}} = 1$ s, Young's modulus $E = 10^7$ Pa, shear modulus $G = 2E/3$ Pa, shear/stretch matrix $\hat{\mathbf{S}} = (4G\hat{A}/3, 4G\hat{A}/3, E\hat{A})$ N, bending/twist matrix $\hat{\mathbf{B}} = \mathrm{diag}(EI_1, EI_2, GI_3)$ Nm^2, dissipation constant $\gamma = 5$ kg m^{-1}s^{-1}), gravity $g = 9.81$ m s^{-2}, forward kinematic friction coefficient $\mu_{\mathrm{k}}^{\mathrm{f}} = 0.519$, friction coefficient ratios $\mu_{\mathrm{k}}^{\mathrm{f}} : \mu_{\mathrm{k}}^{\mathrm{b}} : \mu_{\mathrm{k}}^{\mathrm{r}} = 1 : 1.5 : 2$, ground stiffness and viscous dissipation $k_{\mathrm{w}} = 1000$ kg s^{-2} and $\gamma_{\mathrm{w}} = 10^{-6}$ kg s^{-1}, discretization elements $n = 50$, timestep $\delta t = 1 \cdot 10^{-5}\ T_{\mathrm{m}}$, wavelength $\lambda_{\mathrm{m}} = 0.97L$, torque B-spline coefficients $\beta_{i=0,\ldots,5} = \{0, 17.4, 48.5, 5.4, 14.7, 0\}$ Nm.

Hence, the observed small quantitative differences.

Then to test the above explanation, we reduced shear/twist stiffnesses (i.e softened) of the muscle–tendon groups alone. The rationale being that by softening these elements, the overall group tension might relax, thereby allowing the other modes to be excited. Thus, we softened these modes by 5 times (half of the hardening test). As illustrated in Supplementary Fig. 13, softening has a significant impact with up to ∼60% difference in the observed kinematics. Here we highlight the marked sensitivity of the system response to softening of key components such as the muscle–tendon groups. Additionally, we note that individual twist-softening has an indiscernible effect, thus making shear-softening the primary contributor in altering the kinematics, as can be seen in Supplementary Fig. 13.

In the context of bio-inspired flight, shear should then be an important consideration in

the engineering of feathered ornithopters. This is relevant given the recent focus in aviation on low flight, low speed (relative to aeroplanes) devices, inspired by agile biological flyers (*32, 33*). Thus, as new materials (*34*) that mimic naturally occurring structures are developed and implemented in such devices, it is of use to engineers to know what elements are particularly susceptible to the excitement of unwanted or desired deformation modes.

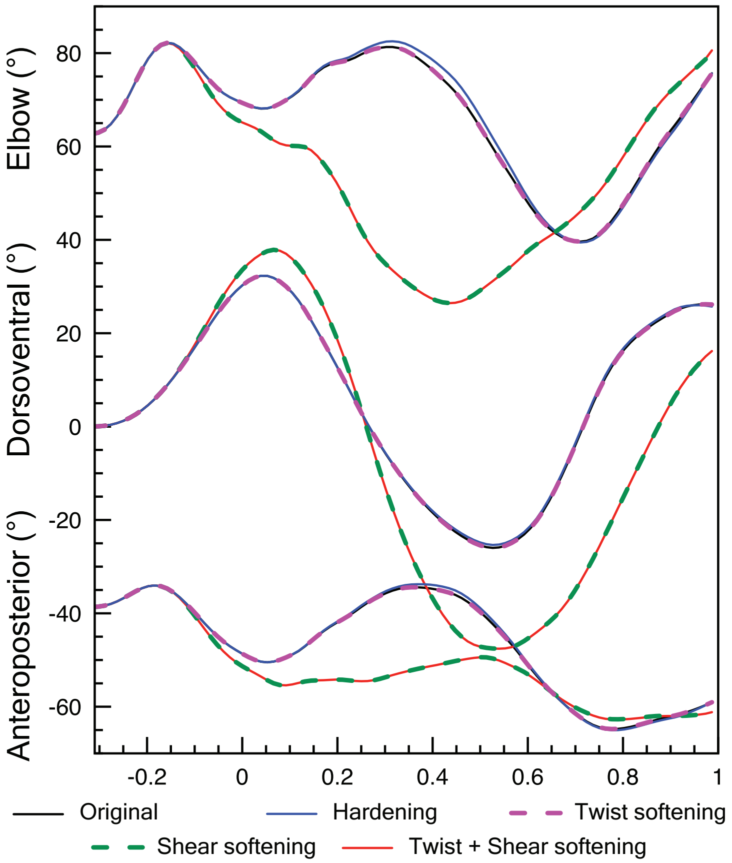

Supplementary Figure 13. Resulting joint angles kinematics of the wing for different hardening/softening configurations as compared with the original. Here, the original wing muscles ($E = 51.2$ kPa, $G = 2E/3$), tendons ($E = 2.6$ GPa, $G = 2E/3$), bones ($E = 38.4$ GPa, $G = 2E/3$) and feathers ($E = 6.4$ MPa, $G = 2E/3$) are all hardened for twist and shear modes by a factor of 10 (i.e. $10G$). For the softening case, these modes are decreased for only the muscles and tendons by a factor of 5 (i.e. $G/5$).

Supplementary Note 6.8 — Summary

The examples in this Supplementary Note provide the physical rationale for enriching the family of elastic rod models with an approach able to capture the dynamics of assemblies of Cosserat rods in which all modes of deformations can be activated. As long as the architectural elements are slender, this method is accurate and robust and allows us to quantitatively test the importance of various deformation modes, either ruling them out or quantifying their impact. Either

way, this will advance the understanding of a given system, potentially providing new design opportunities.

Supplementary Note 7 — Parameters and Derivations

A nomenclature is presented in Supplementary Table 4 including parameters associated with filament interactions and time and spatial discretization schemes in the validation case above, and all three study cases in the main text.

Supplementary Table 4. Nomenclature.

**Notations**	**Parameters**
dt	Time Step
T_{sim}	Simulation Time
$n_{\text{m}}/n_{\text{t}}/n_{\text{b}}/n_{\text{a}}$	Number of Elements in Muscles/Tendons/Bones/Artificial Muscle
$n_{\text{r}}/n_{\text{ba}}$	Discretization Elements in Rachis/Barbs
k	Spherical/Hinge Joint Stiffness
k_{f}	Fixed Joint Stiffness
k_{c}	Rod Collision Stiffness
γ_{c}	Rod Collision Dissipation

Supplementary Note 7.1 — The human elbow joint

- The material properties of all the components in elbow joint can be found on Supplementary Note 2, while the rest of the critical parameters needed for the simulation are given in Supplementary Table 5.

Supplementary Table 5. Parameters for human elbow joint simulation.

**Parameters**	**Values**	**Parameters**	**Values**
dt	5×10^{-8} s	T_{sim}	0.5 s
$n_{\text{m}}/n_{\text{t}}/n_{\text{b}}$	14/7/15–20	k	5×10^{5} kg s^{-2}
k_{f}	5×10^{5} kg s^{-2}	k_{c}	10 kg s^{-2}

- The polynomial fittings for the active muscular force factor $f_{\text{act}}(\eta)$ and the Young's modulus of the parallel elastic component $E_{\text{membrane}}(\eta)$ are written as

$$f_{\text{act}}(\eta) = 6.405\eta^4 - 24.42\eta^3 + 29.64\eta^2 - 12.01\eta + 1.385 \qquad 0.5 \leq \eta \leq 1.6$$
$$E_{\text{membrane}}(\eta) = \begin{cases} 0, & \eta < 1 \\ \frac{F_{\max}}{A} \cdot \left(\frac{3.375\eta^3 - 11.33\eta^2 + 13\eta - 5.05}{\eta - 1}\right), & \eta \geq 1 \end{cases} \tag{3}$$

The muscle's quasi-static force output $F_{\text{qs}}(\eta)$ can thus be expressed based on the fitting equations as

$$F_{\text{qs}}(\eta) = F_{\text{act}} + F_{\text{pass}} = f_{\text{act}}(\eta) \cdot F_{\text{max}} + E_{\text{membrane}}(\eta) \cdot A \cdot (\eta - 1), \tag{4}$$

where the last term takes care of the changes in cross-sectional area of the filament.

- Theoretical solution of $\zeta(v)$ is derived from the Hill's Force–Velocity relation (*16*), which indicates that the dynamic force output $F(v)$ can be related to the isometric quasi-static force F_{qs} via $(F(v) + a)(v + b) = (F_{\text{qs}} + a)b$ where a and b are constants related by $b = av_0/F_{\text{qs}}$ with v_0 denoting the maximum contracting velocity. Assuming $F(v) = F_{\text{qs}} - \zeta(v)v/l_0$ (i.e. quasi-static minus damping force, recalling that $v/l_0 = \dot{\epsilon}$), then:

$$\zeta(v) = \frac{(F_{\text{qs}} + a)F_{\text{qs}}l_0}{av_0 + vF_{\text{qs}}}. \tag{5}$$

  Given the parameters values $F_{\text{qs}} = 1406.3$ N (derived from the torque value in the static test case), $a = 0.16{\cdot}F_{\text{qs}} = 225$ N and $v_0 = 6l_0$ (experimentally determined in (*16*) and (*1*), respectively), we can see how the estimated $\zeta(v)$ determined from our simulations are consistent with theoretical calculations.

- The other parameters needed for the simulation of the arm with artificial muscles, in addition to the ones discussed before in Supplementary Table 3, are given in Supplementary Table 6.

Supplementary Table 6. Parameters for simulation of human elbow joint with artificial muscles.

**Parameters**	**Values**	**Parameters**	**Values**
dt	5×10^{-8} s	T_{sim}	0.7 s
$n_{\text{m}}/n_{\text{t}}/n_{\text{b}}/n_{\text{a}}$	14/7/15–20/108	k	5×10^{5} kg s^{-2}
k_{f}	5×10^{5} kg s^{-2}	k_{c}	10 kg s^{-2}

Supplementary Note 7.2 — Bio-hybrid robots

- The parameters for simulating the bio-hybrid flagella is listed in Supplementary Table 7. Furthermore, the cell cluster is presented as a single filament in the simulation, whose properties are also summarized in the table.
- The dimensions and the simulation parameters of the bio-hybrid walker are presented in Supplementary Table 8. Details of the simulation could be found in literature (*28*).
- In the case of the bio-hybrid walker, we consider bots displacing in a shallow solution that reaches the muscle tissue. As a consequence, the tissue is immersed and suspended in the fluid (due to buoyant forces – density of muscles close to density of liquid). However, the scaffold is mostly exposed to the atmosphere so that we neglect buoyancy and hydrodynamic loads acting on it.

Supplementary Table 7. Parameters for bio-hybrid swimmer simulation.

**Parameters**	**Values**	**Parameters**	**Values**
dt	5×10^{-8} s	T_{sim}	6.5 s
$n_{\text{m}}/n_{\text{b}}$	$2/18$	k	0.38 kg s^{-2}
Cell Length (*35*)	0.107 mm	Cell Radius (*35*)	10 μm
Cell Density[a]	2.6×10^{-4} g mm^{-3}	Cell Young's Modulus (*36*)	30 KPa

[a]Cell density is set to match the aggregate weight of cells given our modeling geometry.

Supplementary Table 8. Dimensions and simulation parameters for bio-hybrid walking bot.

**Parameters**	**Values**	**Parameters**	**Values**
Length/Width/Height	14 mm/7 mm/3.5 mm	Muscle Cross-sectional Area	0.648 mm^2
Scaffold Young's Modulus	319.4 KPa	Scaffold Density	1.12×10^{-3} g mm^{-3}
dt	7.5×10^{-8} s	T_{sim}	10 s
$n_{\text{m}}/n_{\text{b}}$	24/5–14	k	5×10^5 kg s^{-2}
k_{f}	50 kg s^{-2}	k_{c}	10^5 kg s^{-2}
γ_{c}	10^{-6} kg s^{-1}		

- The muscle contractile stress $\sigma_{\text{m}} = \frac{1}{\gamma}(\frac{F_{\text{m}}}{A_{\text{m}}} + \frac{E_{\text{m}}\epsilon}{1-\epsilon}) = 3.706$ kPa, where ratio of active-to-total muscle cross section area $\gamma = 1$, muscle output force $F_{\text{m}} = 1.4$ mN, muscle total area $A_{\text{m}} = 0.648\text{mm}^2$ and muscle strain $\epsilon = 0.134$ are characterized from experiments (*28*). Muscle's Young's modulus $E_{\text{m}} = 10$ kPa is found in Supplementary Table 1.

Supplementary Note 7.3 — Soft, limbless, slithering robots

- Biomechanical properties of muscles and tendons are adopted from the previous case. Parameters for simulating the soft snake robot are presented in Supplementary Table 9.

Supplementary Table 9. Parameters for soft snake bot simulation.

**Parameters**	**Values**	**Parameters**	**Values**
dt	2×10^{-8} s	T_{sim}	5 s
$n_{\text{m}}/n_{\text{t}}/n_{\text{b}}$	4/5/100	k	5×10^9 kg s^{-2}
Ground Stiffness	1 kg s^{-2}	Ground Damping	10^{-6} kg s^{-1}
Kinetic Friction Coefficients Forward/Sideways/Backward			1/2/1.5
Static Friction Coefficients Forward/Sideways/Backward			2/4/3

Supplementary Note 7.4 — Feathered wings

- Parameters for simulating the feathered wing are listed in Supplementary Table 10.

Supplementary Table 10. Simulation parameters for feathered wing.

**Parameters**	**Values**	**Parameters**	**Values**
dt	5×10^{-8} s	T_{sim}	0.51 s
$n_{\text{m}}/n_{\text{b}}$	7/10–12	$n_{\text{r}}/n_{\text{ba}}$	8/4
k	5×10^{5} kg s^{-2}	k_{f}	8×10^{4} kg s^{-2}

- Muscular forces for biceps (BC), scapulotriceps (ST), pectoralis (PECT), and supracoracoideus (SUP) during initiation phase for simulation time $0 < t < 0.125$ s in preparation for downstroke configuration are prescribed as

$$\text{Activity}(t) = \begin{cases} 0.0, & 0 < t < 0.1250 \text{ s}, \quad \text{BC} \\ 11.06 \cdot \sin(16\pi t), & 0 < t < 0.0625 \text{ s}, \quad \text{ST} \\ 0.0, & 0 < t < 0.1250 \text{ s}, \quad \text{PECT} \\ 6.14 \cdot \sin(8\pi t), & 0 < t < 0.1250 \text{ s}, \quad \text{SUP} \end{cases} \tag{6}$$

- Different muscular forces for the full stroke cycle of takeoff wing motion based on EMG signals from (*37*) are prescribed as

$$\text{Activity}_{\text{BC}}(t) = \begin{cases} 9.83 \cdot \sin\left(\dfrac{2\pi}{0.6579 \cdot T}\left[t + 0.0395 \cdot T\right]\right), & 0 < t < 0.1250\,T \\ 9.83 \cdot \left(1 - 0.7 \cdot \left[\dfrac{t - 0.125 \cdot T}{0.1645 \cdot T}\right]\right), & 0.1250\,T < t < 0.2895\,T \\ 2.95 \cdot \sin\left(\dfrac{2\pi}{0.6579 \cdot T} \cdot \left[t - 0.125 \cdot T\right]\right), & 0.2895\,T < t < 0.4539\,T \\ 0, & 0.4539\,T < t < 0.9605\,T \\ 9.83 \cdot \sin\left(\dfrac{2\pi}{0.6579 \cdot T} \cdot \left[t - 0.9605 \cdot T\right]\right), & 0.9605\,T < t < T \end{cases} \tag{7}$$

$$\text{Activity}_{\text{ST}}(t) = \begin{cases} 0, & 0 < t < 0.3816\,T \\ 3.28 \cdot \sin\left(\dfrac{2\pi}{0.6579 \cdot T}\left[t - 0.3816 \cdot T\right]\right), & 0.3816\,T < t < 0.5461\,T \\ 3.28 \cdot \left(1 - 0.35 \cdot \left[\dfrac{t - 0.5461 \cdot T}{0.1645 \cdot T}\right]\right), & 0.5461\,T < t < 0.7105\,T \\ 2.13 \cdot \sin\left(\dfrac{2\pi}{0.6579 \cdot T} \cdot \left[t - 0.5461 \cdot T\right]\right), & 0.7105\,T < t < 0.8750\,T \\ 0, & 0.8750\,T < t < T \end{cases} \tag{8}$$

$$\text{Activity}_{\text{PECT}}(t) = \begin{cases} 7.37 \cdot \sin\left(\dfrac{2\pi}{0.6579 \cdot T}\left[t + 0.0395 \cdot T\right]\right), & 0 < t < 0.1250\,T \\ 7.37 \cdot \left(1 - 0.5 \cdot \left[\dfrac{t - 0.125 \cdot T}{0.1645 \cdot T}\right]\right), & 0.1250\,T < t < 0.2895\,T \\ 3.68 \cdot \sin\left(\dfrac{2\pi}{0.6579 \cdot T} \cdot \left[t - 0.125 \cdot T\right]\right), & 0.2895\,T < t < 0.4539\,T \\ 0, & 0.4539\,T < t < 0.9605\,T \\ 7.37 \cdot \sin\left(\dfrac{2\pi}{0.6579 \cdot T} \cdot \left[t - 0.9605 \cdot T\right]\right), & 0.9605\,T < t < T \end{cases} \tag{9}$$

$$\text{Activity}_{\text{SUP}}(t) = \begin{cases} 0, & 0 < t < 0.4079\,T \\ 7.37 \cdot \sin\left(\dfrac{2\pi}{0.6579 \cdot T}\left[t - 0.4079 \cdot T\right]\right), & 0.4079\,T < t < 0.5724\,T \\ 7.37 \cdot \left(1 - 0.6 \cdot \left[\dfrac{t - 0.5724 \cdot T}{0.1645 \cdot T}\right]\right), & 0.5724\,T < t < 0.7368\,T \\ 2.95 \cdot \sin\left(\dfrac{2\pi}{0.6579 \cdot T} \cdot \left[t - 0.5724 \cdot T\right]\right), & 0.7368\,T < t < 0.9013\,T \\ 0, & 0.9013\,T < t < T \end{cases} \tag{10}$$

where $T = 0.38$ s is the time period.

References

1. Winter, D. A. *Biomechanics and motor control of human movement Ch. 4, 9* (John Wiley & Sons, 2009).

2. van den Bogert, A. J., Gerritsen, K. G. & Cole, G. K. Human muscle modelling from a user's perspective. *Journal of Electromyography and Kinesiology* **8**, 119–124 (1998).

3. Cofer, D. *et al.* Animatlab: a 3d graphics environment for neuromechanical simulations. *Journal of Neuroscience Methods* **187**, 280–288 (2010).

4. Conwit, R. *et al.* The relationship of motor unit size, firing rate and force. *Clinical Neurophysiology* **110**, 1270–1275 (1999).

5. Buchthal, F. & Schmalbruch, H. Motor unit of mammalian muscle. *Physiological Reviews* **60**, 90–142 (1980).

6. McComas, A., Fawcett, P. R. W., Campbell, M. & Sica, R. Electrophysiological estimation of the number of motor units within a human muscle. *Journal of Neurology, Neurosurgery & Psychiatry* **34**, 121–131 (1971).

7. Milner-Brown, H., Stein, R. & Yemm, R. The orderly recruitment of human motor units during voluntary isometric contractions. *The Journal of Physiology* **230**, 359–370 (1973).

8. Brown, W. F., Strong, M. J. & Snow, R. Methods for estimating numbers of motor units in biceps-brachialis muscles and losses of motor units with aging. *Muscle & Nerve* **11**, 423–432 (1988).

9. Klein, C. S., Marsh, G. D., Petrella, R. J. & Rice, C. L. Muscle fiber number in the biceps brachii muscle of young and old men. *Muscle & Nerve* **28**, 62–68 (2003).

10. Kot, B., Zhang, Z., Lee, A., Leung, V. & Fu, S. Elastic modulus of muscle and tendon with shear wave ultrasound elastography: variations with different technical settings. *PLOS One* **7**, e44348 (2012).

11. Méndez, J. Density and composition of mammalian muscle. *Metabolism* **9**, 184–188 (1960).

12. Rho, J.-Y., Hobatho, M. & Ashman, R. Relations of mechanical properties to density and ct numbers in human bone. *Medical Engineering & Physics* **17**, 347–355 (1995).

13. Hashemi, J., Chandrashekar, N. & Slauterbeck, J. The mechanical properties of the human patellar tendon are correlated to its mass density and are independent of sex. *Clinical Biomechanics* **20**, 645–652 (2005).

14. Rubenson, J., Pires, N., Loi, H., Pinniger, G. & Shannon, D. On the ascent: the soleus operating length is conserved to the ascending limb of the force–length curve across gait mechanics in humans. *Journal of Experimental Biology* **215**, 3539–3551 (2012).

15. Gauthier, A., Davenne, D., Martin, A. & Van Hoecke, J. Time of day effects on isometric and isokinetic torque developed during elbow flexion in humans. *European Journal of Applied Physiology* **84**, 249–252 (2001).

16. Hill, A. The heat of shortening and the dynamic constants of muscle. *Proceedings of the Royal Society of London B: Biological Sciences* **126**, 136–195 (1938).

17. Cox, R. The motion of long slender bodies in a viscous fluid part 1. general theory. *Journal of Fluid Mechanics* **44**, 791–810 (1970).

18. Yanoviak, S. P., Munk, Y., Kaspari, M. & Dudley, R. Aerial manoeuvrability in wingless gliding ants (*Cephalotes atratus*). *Proceedings of the Royal Society B: Biological Sciences* **277**, 2199–2204 (2010).

19. Bachmann, T., Emmerlich, J., Baumgartner, W., Schneider, J. & Wagner, H. Flexural stiffness of feather shafts: geometry rules over material properties. *Journal of Experimental Biology* **215**, 405–415 (2012).

20. Bachmann, T. *et al.* Morphometric characterisation of wing feathers of the barn owl *Tyto alba pratincola* and the pigeon *Columba livia*. *Frontiers in Zoology* **4**, 23 (2007).

21. Sullivan, T. N. *et al.* A lightweight, biological structure with tailored stiffness: The feather vane. *Acta Biomaterialia* **41**, 27–39 (2016).

22. Perez, C. R., Moye, J. K. & Pritsos, C. A. Estimating the surface area of birds: using the homing pigeon (*Columba livia*) as a model. *Biology Open* **3**, 486–488 (2014).

23. Munson, B. R., Young, D. F. & Okiishi, T. H. Fundamentals of fluid mechanics. *Oceanographic Literature Review* **10**, 831 (1995).

24. Haines, C. *et al.* Artificial muscles from fishing line and sewing thread. *Science* **343**, 868–872 (2014).

25. Haines, C. *et al.* New twist on artificial muscles. *Proceedings of the National Academy of Sciences* **113**, 11709–11716 (2016).

26. Charles, N., Gazzola, M. & Mahadevan, L. Topology, geometry and mechanics of strongly stretched and twisted filaments (2019). Preprint at https://arxiv.org/abs/1905.00796.

27. Williams, B., Anand, S., Rajagopalan, J. & Saif, M. A self-propelled biohybrid swimmer at low reynolds number. *Nature Communications* **5**, 3081 (2014).

28. Pagan-Diaz, G. J. *et al.* Simulation and fabrication of stronger, larger, and faster walking biohybrid machines. *Advanced Functional Materials* **28**, 1801145 (2018).

29. Hu, D., Nirody, J., Scott, T. & Shelley, M. The mechanics of slithering locomotion. *Proceedings of the National Academy of Sciences* **106**, 10081–10085 (2009).

30. Rafsanjani, A., Zhang, Y., Liu, B., Rubinstein, S. M. & Bertoldi, K. Kirigami skins make a simple soft actuator crawl. *Science Robotics* **3**, eaar7555 (2018).

31. Gazzola, M., Dudte, L. H., McCormick, A. G. & Mahadevan, L. Forward and inverse problems in the mechanics of soft filaments. *Royal Society Open Science* **5**, 171628 (2018).

32. Lentink, D., Jongerius, S. R. & Bradshaw, N. L. The scalable design of flapping micro-air vehicles inspired by insect flight. In *Flying Insects and Robots*, 185–205 (Springer, 2009).

33. Wissa, A. *et al.* Free flight testing and performance evaluation of a passively morphing ornithopter. *International Journal of Micro Air Vehicles* **7**, 21–40 (2015).

34. Malakooti, M. H., Zhou, Z., Spears, J. H., Shankwitz, T. J. & Sodano, H. A. Biomimetic nanostructured interfaces for hierarchical composites. *Advanced Materials Interfaces* **3**, 1500404 (2016).

35. Du, Y., Plante, E., Janicki, J. S. & Brower, G. L. Temporal evaluation of cardiac myocyte hypertrophy and hyperplasia in male rats secondary to chronic volume overload. *The American Journal of Pathology* **177**, 1155–1163 (2010).

36. Jacot, J. G., Martin, J. C. & Hunt, D. L. Mechanobiology of cardiomyocyte development. *Journal of Biomechanics* **43**, 93–98 (2010).

37. Robertson, A. M. B. & Biewener, A. A. Muscle function during takeoff and landing flight in the pigeon (*Columba livia*). *Journal of Experimental Biology* **215**, 4104–4114 (2012).
