# Supplementary material for: Modeling and simulation of complex dynamic musculoskeletal architectures
Source: Nat Commun. 2019 Oct 23;10:4825. doi: 10.1038/s41467-019-12759-5 (PMC6811595; doi:10.1038/s41467-019-12759-5)
Supplement: Supplementary file 10 — Description of Additional Supplementary Files [file 41467_2019_12759_MOESM10_ESM.pdf]

Title: Supplementary Movie 1
Description: Simulation result of an elbow joint performing isokinetic joint flexion. Humerus, ulna and radius are modeled with stiff filaments in purple. Tendons are modeled with tapering filaments in yellow. Two heads of biceps consisting of 36 filaments are performing maximum voluntary contraction. Boundary conditions are applied to ensure a constant angular velocity.

Title: Supplementary Movie 2
Description: Comparison between the original left and the optimal right swimming robot designs. Actuation frequencies for both cases are 3.6 Hz.

Title: Supplementary Movie 3
Simulation of walking robot with actuation frequency 2 Hz.

Title: Supplementary Movie 4
Description: Slithering motion of the musculoskeletal snake model with four muscle groups. The muscle group arrangements are optimized for the fastest forward velocity. Color of the muscle groups indicates level of muscle contraction.

Title: Supplementary Movie 5
Description: The front view of a pair of wings consisting of 6342 filaments, where green filaments indicate the feathers. An initiation process lifts the wings from flat position and is followed by a single power downstroke and upstroke during the takeoff stage.

Title: Supplementary Movie 6
Description: The lateral view of the same motion.

Title: Supplementary Movie 7
Description: Comparison between a healthy muscle, an injured one and one enhanced with an artificial muscle in lifting a weight.

Title: Supplementary Movie 8
Description: Comparison between the original and shear-hardened snake performing a task of overcoming a sloped barrier.
